# Anti-Tumour Efficacy of Capecitabine in a Genetically Engineered Mouse Model of Pancreatic Cancer

**DOI:** 10.1371/journal.pone.0067330

**Published:** 2013-06-28

**Authors:** Aurélie Courtin, Frances M. Richards, Tashinga E. Bapiro, Jo L. Bramhall, Albrecht Neesse, Natalie Cook, Ben-Fillippo Krippendorff, David A. Tuveson, Duncan I. Jodrell

**Affiliations:** 1 Pharmacology and Drug Development Group, Cancer Research UK Cambridge Research Institute, Cambridge, United Kingdom; 2 Tumour Modelling and Experimental Medicine Group, Cancer Research UK Cambridge Research Institute, Cambridge, United Kingdom; 3 University of Cambridge Department of Oncology, Cambridge, United Kingdom, Cambridge, United Kingdom; Technische Universität München, Germany

## Abstract

Capecitabine (CAP) is a 5-FU pro-drug approved for the treatment of several cancers and it is used in combination with gemcitabine (GEM) in the treatment of patients with pancreatic adenocarcinoma (PDAC). However, limited pre-clinical data of the effects of CAP in PDAC are available to support the use of the GEMCAP combination in clinic. Therefore, we investigated the pharmacokinetics and the efficacy of CAP as a single agent first and then in combination with GEM to assess the utility of the GEMCAP therapy in clinic. Using a model of spontaneous PDAC occurring in Kras^G12D^; p53^R172H^; Pdx1-Cre (KPC) mice and subcutaneous allografts of a KPC PDAC-derived cell line (K8484), we showed that CAP achieved tumour concentrations (∼25 µM) of 5-FU in both models, as a single agent, and induced survival similar to GEM in KPC mice, suggesting similar efficacy. *In vitro* studies performed in K8484 cells as well as in human pancreatic cell lines showed an additive effect of the GEMCAP combination however, it increased toxicity *in vivo* and no benefit of a tolerable GEMCAP combination was identified in the allograft model when compared to GEM alone. Our work provides pre-clinical evidence of 5-FU delivery to tumours and anti-tumour efficacy following oral CAP administration that was similar to effects of GEM. Nevertheless, the GEMCAP combination does not improve the therapeutic index compared to GEM alone. These data suggest that CAP could be considered as an alternative to GEM in future, rationally designed, combination treatment strategies for advanced pancreatic cancer.

## Introduction

Pancreatic ductal adenocarcinoma (PDAC) is the fourth leading cause of cancer-related deaths in industrialised countries. Overall 5 year survival rate is less than 5% [1]. The high degree of mortality of PDAC is attributable to the lack of early detection methods and the poor efficacy of existing therapies. Gemcitabine (GEM) is the standard therapy, but the median survival time remains only 5–7 months in patients with advanced disease [2]. Therefore, more effective treatment strategies are required.

Capecitabine (Xeloda®; Hoffmann La Roche) is an orally administered fluoropyrimidine carbamate, metabolised in liver and tumour by carboxylesterases and cytidine deaminase to 5′-deoxy-5-fluorocytidine (5′-DFCR) and 5′-deoxy-5-fluorouridine (5′-DFUR) respectively. The final step of the activation of capecitabine (CAP), conversion of 5′-DFUR to cytotoxic 5-fluorouracil (5-FU), is mediated by thymidine phosphorylase [3]–[5], which is expressed more highly in neoplastic than normal tissue, making CAP more tumour specific than 5-FU [5], [6]. Anti-tumour efficacy of CAP has been shown in numerous studies using human cancer xenograft models of breast, colon, gastric, cervical, bladder ovarian and prostate cancer (see for review [7]) but only one study has been reported in a pancreas model [8] and this was in an atypical KRAS wild type pancreatic cancer cell xenograft. In the clinic, CAP is approved by the FDA as first line single agent therapy in patients with metastatic colorectal cancer and for metastatic breast cancer as a single agent or in combination with docetaxel after failure of prior anthracyline-based chemotherapy. In patients with completely resected pancreatic cancer, it has been shown that combined intravenous bolus of 5-fluorouracil and folinic acid (FUFA) is an active adjuvant therapy and the use of FUFA is equivalent to GEM when overall survival is the end-point [9], [10]. In advanced PDAC, particularly in the UK, CAP has replaced FUFA and is used in combination with GEM (GEMCAP), based on the results of the meta-analysis performed by Heinemann and al. showing a modest but significant survival benefit from the combination of GEM with a fluoropyrimidine and especially with CAP [2]. A recent clinical study confirmed the benefit of GEMCAP in unselected patients with advanced PDAC [11].

In view of the limited pre-clinical data using CAP in PDAC, *in vivo* studies were undertaken to evaluate CAP in a genetically engineered mouse model of the disease. Kras^G12D^; p53^R172H^; Pdx1-Cre (KPC) mice conditionally express endogenous mutant Kras and p53 alleles in pancreatic cells [12] and develop pancreatic tumours, which recapitulate the pathophysiological aspects and the molecular features of human PDAC [13]. We also used an allograft of a pancreatic cancer cell line (K8484) isolated from a KPC PDAC. Pharmacokinetic and efficacy studies were performed using single agent CAP and the combination of GEM and CAP. Studies from the literature suggested an association between cytidine deaminase (CDA) enzyme activity and the risk of toxicity in patients receiving GEM or CAP-based therapy [14], [15]. CDA is involved in the activation of CAP through the deamination of dFCR into dFUR but is conversely responsible for the deamination of GEM into its inactive metabolite dFdU [4], [5], [16], [17]. Because of the toxicity we observed with the GEMCAP combination we quantified CDA enzyme activity in the tumour tissue.

## Materials and Methods

### Mouse Strains

KPC mice develop advanced PDAC from 2 to 3 months and have a shortened median survival of approximately 5 months [12], [18]. Their control littermates, Kras; p53^R172H^; Pdx1-Cre (PC) mice were also used in this study to transplant subcutaneously the K8484 cell line. Thus, all the experiments were performed using mice from the same mixed 129/SvJal/C57BL/6 genetic background. All experiments were carried out in accordance with the UK Animals (Scientific Procedures) Act 1986 with approval from the local Animal Ethics Committee, and following the 2010 guidelines from the United Kingdom Coordinating Committee on Cancer Research [19].

### Cell Lines

The K8484 cell line was established from a KPC PDAC tumour by Olive et al. [18], [20]. Cells were grown in DMEM medium (Life Technologies) supplemented with 5% fetal bovine serum (FBS). The human pancreatic cancer cell lines Panc-1 and MiaPaCa-2 were obtained from ECACC (Salisbury, UK) and grown in DMEM with 10% FBS. The identity of all human cell lines were verified by STR genotyping and tested negative for mycoplasma.

### Cytotoxicity Assay

Drug cytotoxicity *in vitro* was assessed by the means of Sulforhodamine B colorimetric (SRB) assay. Cells were plated in a 96 well plate and dosed with a range of concentrations of 5-FU (0.03 µM to 30 µM) in rows and gemcitabine (3×10^−4^ µM to 0.3 µM) in columns, giving a grid of 8×8 concentration combinations. After 72 h of incubation at 37°C, cells were then fixed (3% trichloroacetic acid, 90 minutes, 4°C), washed in water and stained with a 0.057% SRB (Sigma) solution in acetic acid (w/v) for 30 minutes. The plates were washed (1% acetic acid, 4 times), and the protein-bound dye was dissolved in a 10 mM Tris base solution (pH 10.5). Fluorescence was measured using Tecan Infinite M200 plate-reader (excitation 488 nm, emission 585 nm). The % Growth Inhibition (GI) compared to solvent control-treated cells was calculated for each drug concentration combination.

The effect of the combination was evaluated using the Bliss Independance model [21], [22] according to the protocol we have described previously [23]. Briefly, an additivity model was built based on single agent data from 5-FU and GEM. GI values obtained from this model were then subtracted from the experimental values to identify regions of synergy and antagonism. Negative numbers show less than additive effects (yellow to red colour) and positive numbers show greater than additive effect (green to blue).

### Drug preparation

Capecitabine powder (Sequoia Research) was resuspended in a 40 mM citrate buffer and 5% gum Arabic at 100 mg/ml and administered by oral gavage. Gemcitabine hydrochloride (Tocris Bioscience) was dissolved in saline at 20 mg/ml and administered by intraperitoneal injection.

### Pharmacokinetics and Efficacy Studies in K8484 Allograft Model

Bilateral K8484 allografts were obtained by subcutaneous injection of 10^6^ cells per flank. For pharmacokinetics studies, mice bearing allograft tumour were treated with a single dose of CAP at 755 mg/kg and samples were collected 10 min, 20 min, 40 min, 1 h, 2 h and 4 h after, three animals per time point. Blood was collected in Lithium heparin, and plasma isolated and stored at −80°C. Liver and tumours were removed and snap-frozen in liquid nitrogen.

In CAP efficacy studies, mice were treated with CAP at 755 mg/kg or vehicle for 5 consecutive days per week for 3 weeks. In GEMCAP combination efficacy study, mice received gemcitabine doses at 75 mg/kg, every 3 days alone or in combination with oral CAP doses at 539 mg/kg given 5 days per week. Tumours were measured using by callipers. Tumour volume was calculated as length×width^2^×π/6.

### Efficacy and Survival Study in KPC Mouse Model

The enrolment of KPC mice in study was based on tumour size, measured by ultrasound in an axial orientation. Mice with mean PDAC tumour diameters of 6–9 mm were enrolled. For the short term efficacy study, mice were treated with CAP at 755 mg/kg or vehicle for 7 consecutive days. During treatment, mice were imaged twice by high resolution ultrasound imaging using the Vevo 770 System (Visual Sonics, Inc) [24] and tumour volumes were quantified [18]. On day 7, mice were killed 2 h after the CAP dose. Plasma, tumour and liver were collected as above. For the survival study, mice were enrolled, and imaged every 3 days whilst treating with CAP at 755 mg/kg for 5 consecutive days per week or with GEM at 100 mg/kg every 3 days until the endpoint criteria were reached. These included the development of abdominal ascites, severe cachexia, significant weight loss (approaching 20%) of initial weight or extreme weakness or inactivity.

### Immunohistochemistry

Formalin-fixed, paraffin embedded tissue sections were stained using phospho-histone H3 antibody (Upstate, #06-570) and detected using DAB Peroxidase Substrate (Vector Labs). Staining was imaged and quantified using the Ariol system (Leica Microsystems). A minimum of 3 fields per tumour were quantified. PH3 positive cells were defined as those having positively staining condensed chromatin.

### GEMCAP Combination Tolerance Studies

PC mice bearing K8484 cell allografts were treated with GEM at 100 mg/kg or 75 mg/kg every 3 days alone or in combination with CAP at 755 mg/kg or 539 mg/kg or 378 mg/kg, 5 consecutive days per week and were killed if any clinical signs approached the permitted limits (which included diarrhoea, haemorrhage and weight loss approaching 20%). Two animals per dose were used. Tumour response was assessed by daily measurement of the tumours with callipers.

### Determination of Capecitabine and Metabolite Concentrations in Plasma, Liver and Tumours

CAP, DFCR, DFUR and 5FU were determined both in plasma and tissues using a modified version of the protocol previously published [25]. Briefly, tissue samples were homogenised using a Precellys 24 homogeniser with small ball bearings (Kit Precellys MK28-R) for 2×50 seconds at 6 000 rpm in ice-cold 50∶50 acetonitrile:water (v/v) containing 25 µg/ml tetrahydrouridine (Promega) to make a final concentration of 50 mg tissue per ml. Fifty µl of homogenate was transferred to a clean tube prespiked with 200 µl of ice-cold acetonitrile containing 50 ng/ml stable labelled (SIL) internal standards of all 4 analytes (Toronto Research Chemicals). After centrifugation at 20,000 g for 5 minutes the supernatant was evaporated to dryness, resuspended in 100 µL of water and injected onto the LC-MS/MS. For plasma samples, 50 µL of plasma was precipitated with 150 µL of acetonitrile containing 50 ng/ml stable labelled internal standards and processed similarly.

Detection was achieved by LC-MS/MS using a Thermo TSQ Vantage mass spectrometer with a HESI-II probe operated in positive and negative mode at a spray voltage of 3 kV and vaporizer temperature of 325°C. Detection of the ions was performed in the multiple reaction monitoring mode, specific for each compound. Concentrations of capecitabine and metabolites in samples were determined by comparison against calibration lines constructed using authentic reference standards.

### CDA Enzyme Activity

To prepare crude enzyme matrix, tumour tissue was homogenised in 500 µL 0.1 M Tris-HCl pH8 buffer for 2×50 seconds at 6000 rpm using a Precelly homogeniser. Tumour lysates were then centrifuged for 10 min at 14500 rpm at 4°C, supernatants decanted and the protein content was measured using the DC-BIORAD protein assay.

For CDA enzyme activity assay, GEM stock solutions were prepared in water. For each reaction, 20 µL of tumour enzyme extract was mixed with 170 µL 0.1 M Tris-HCl, 50 mM β-mercaptoethanol and 10 µL of the appropriate gemcitabine stock solution to make final GEM substrate concentrations of 50 µM to 5000 µM. Samples were incubated at 37°C for 30 minutes, the reaction was stopped by adding 600 µL of acetonitrile and 50 µL of internal standard dFdU-^13^C, ^15^N_2_ was added to each sample. The same protocol was used to prepare a dFdU standard curve ranging from 2.5 ng/mL to 5000 ng/mL as well as High (3500 ng/mL), medium (200 ng/mL) and low (37.5 ng/mL) quality control dFdU standards.

All samples were mixed and centrifuged at 5000 rpm for 10 minutes at 4°C. 200 µL of supernatant were transferred in a 96 well plate and evaporated to dryness. Samples were then reconstituted in 200 µL water, vortexed for 5 minutes and the dFdU was quantified by LC-MS/MS and normalised to the total protein concentrations.

The *in vitro* competition assay was performed on an enzyme extract from an untreated allograft tumour using the protocol described above. The enzyme extract was mixed with a fixed concentration of 1800 µM GEM, corresponding to the Km of CDA in this enzyme extract, and ranging doses of DFCR (0 to 1200 µM) as competitor. The dFdU converted from GEM was quantified by LC-MS/MS.

### Statistical Analysis

Statistical analyses were carried out using GraphPad Prism version 5.0. Significance of the differences in CAP anti-tumour efficacy, CDA Vmax and Km for CAP versus vehicle was determined using an unpaired t-test. In tolerance and GEMCAP combination studies, distinction in tumour growths were analysed using a one way ANOVA followed by a Newman-Keuls multiple comparisons post-test.

## Results

### Pharmacokinetics and Pharmacodynamics of CAP and its Metabolites in Mouse Tissues

**Figure 1:**
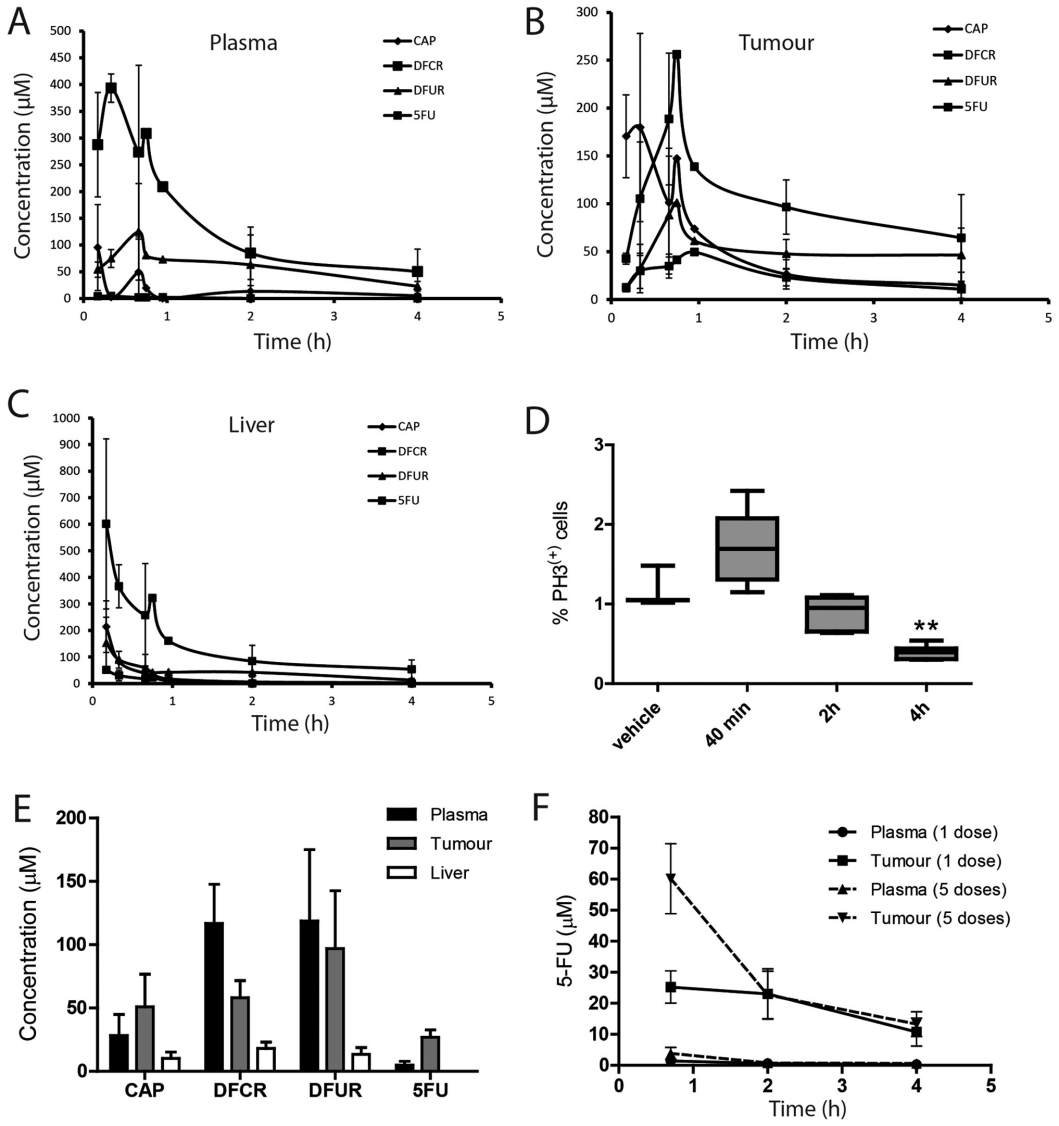
Pharmacokinetic and pharmacodynamics profiles of CAP in mouse tissues. Metabolite concentrations in plasma (A), K8484 allograft tumour homogenates (B) and liver homogenates (C) after a single dose of CAP at 755 mg/kg. Mean ± SD (n = 3 except for 45 min and 1 h where n = 2). (D) Immunohistochemistry for phospho-histone H3 (PH3) quantified in KPC allograft tumours at different time points after a single 755 mg/kg CAP dose. Mean ± SEM of at least three tumours (**p<0.01). (E) Metabolite concentrations in KPC mouse tissues 2 h after the last of 7 consecutive doses of CAP at 755 mg/kg, Mean and SEM of 6 mice. (F) 5-FU concentrations 2 h after a single dose (solid line) or five consecutive doses (dashed line) of CAP (755 mg/kg) given to mice bearing K8484 allograft tumours.

To investigate the pharmacokinetics of CAP and its metabolites, we analysed by mass spectrometry the homogenates of plasma, tumour and liver from mice bearing K8484 allograft tumour collected at different time points after a single dose of CAP given orally at 755 mg/kg (2.1 mmol/kg/day). This dose is the human equivalent dose, determined according to the method of Reagan-Shaw et al [26]. CAP, DFCR and DFUR were found in the 3 tissues at each time point (Figure 1). Plasma C_max_ for DFCR was 393±27 µM (20 mins) and for DFUR 125±90 µM (40 mins) (Figure 1A). In tumour tissue, DFCR and DFUR C_max_ were 256±7 µM and 101±7 µM respectively 45 min after CAP administration and fell to 64.4±45.3 µM and 46.4±28.1 µM after 4 h (Figure 1B). In liver, C_max_ for all the metabolites were reached 10 min after dosing and then decreased progressively (Figure 1C). The concentrations of liver DFCR were higher than in tumour from the same mice and the liver DFUR concentrations were lower, consistent with the previously reported distributions of CES and CDA in tissues [5]. Intra-tumoural concentrations of 5-FU increased progressively from 12.7±3.7 µM after 10 min to 49.5 µM after 1 h and fell to 10.7±3.4 µM after 4 h (Figure 1B). As our *in vitro* experiments on K8484 cells showed an IC_50_ for 5-FU of 2.56±0.55 µM (data not shown), these *in vivo* results suggest that an oral CAP dosing delivers a therapeutically effective dose to the allograft tumours. 5-FU levels were more than 30 fold lower in plasma (1.9±0.5 µM at 40 min and 0.3±0.2 µM after 4 h) than in tumour and were below the limit of quantification in the liver from 2 h (Figure 1C). A pharmacodynamic study was also performed to determine the effect of a single 755 mg/kg CAP dose on proliferation in K8484 allograft tumours. Tumours were collected 40 min, 2 h and 4 h after dosing and immunohistochemistry for phospho-histone H3 (PH3) was quantified. PH3 staining was significantly decreased 4 h after dosing (P<0.01; Figure 1D) compared to control, revealing a decrease in mitotic cell number following CAP treatment.

It has been shown previously that *in situ* KPC PDAC tumours are less sensitive to GEM than KPC allografts despite their identical *Kras* and *p53* genotypes, and this difference in sensitivity was attributed to limited drug delivery to the KPC tumours [18]. Therefore, we analysed the amount of CAP and metabolites in tissues from KPC tumour bearing mice after seven days of CAP treatment (755 mg/kg). In plasma and liver CAP, DFCR, DFUR and 5-FU concentrations were comparable between KPC mice and the allograft hosts 2 h after the last dose (Figure 1E). In KPC PDAC tumours, 5-FU concentrations were 26.9±14.3 µM (Figure 1E) compared to 23.0±8.1 µM in KPC allograft tumours, 2 h after a single CAP dose (Figure 1B). Because of the difference in study protocol (7 doses in KPC mice versus single dose in the K8484 allografts), we also compared the data with those from an independent PK study performed after 5 daily doses of CAP administered in mice bearing K8484 allografts. In this study 5-FU concentrations in tumour 2 h after the fifth consecutive dose of CAP was 22.7±7.7 µM (Figure 1F), equivalent to the 5-FU concentration after a single dose in allograft tumour (Figure 1B) or after 7 consecutive doses in KPC tumours (Figure 1E). These data suggest that multiple doses of CAP did not lead to 5-FU accumulation in tumour. These results show that oral CAP delivered a similar amount of 5-FU to the *in situ* PDAC tumours and to the allograft tumours.

### CAP Anti-tumour Efficacy in Pancreatic Tumours

**Figure 2:**
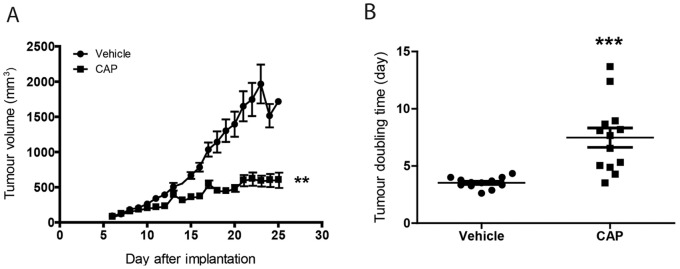
Anti-tumour effect of capecitabine in KPC allograft tumours. (A) Capecitabine efficacy in mice bearing K8484 allograft tumours. Animals received 755 mg/kg daily for 5 consecutive days per week for 3 weeks. Tumour volumes are represented as the mean ± SEM of 12 vehicle- and 13 CAP-treated mice (**p<0.01). (B) The tumour doubling time for each tumour (***p<0.001).

We first studied the effect of CAP on the growth of K8484 allograft tumours. Mice were dosed with vehicle or CAP at 755 mg/kg for 5 days per week. After 3 weeks, vehicle-treated mice exhibited an average tumour volume of 1840±201 mm^3^ compared to 629±86 mm^3^ in CAP-treated mice (Figure 2A), with a significant increase in tumour doubling time from 3.5±0.5 days in vehicle to 7.5±3.0 days in CAP-treated mice (P = 0.0002; Figure 2B).

**Figure 3:**
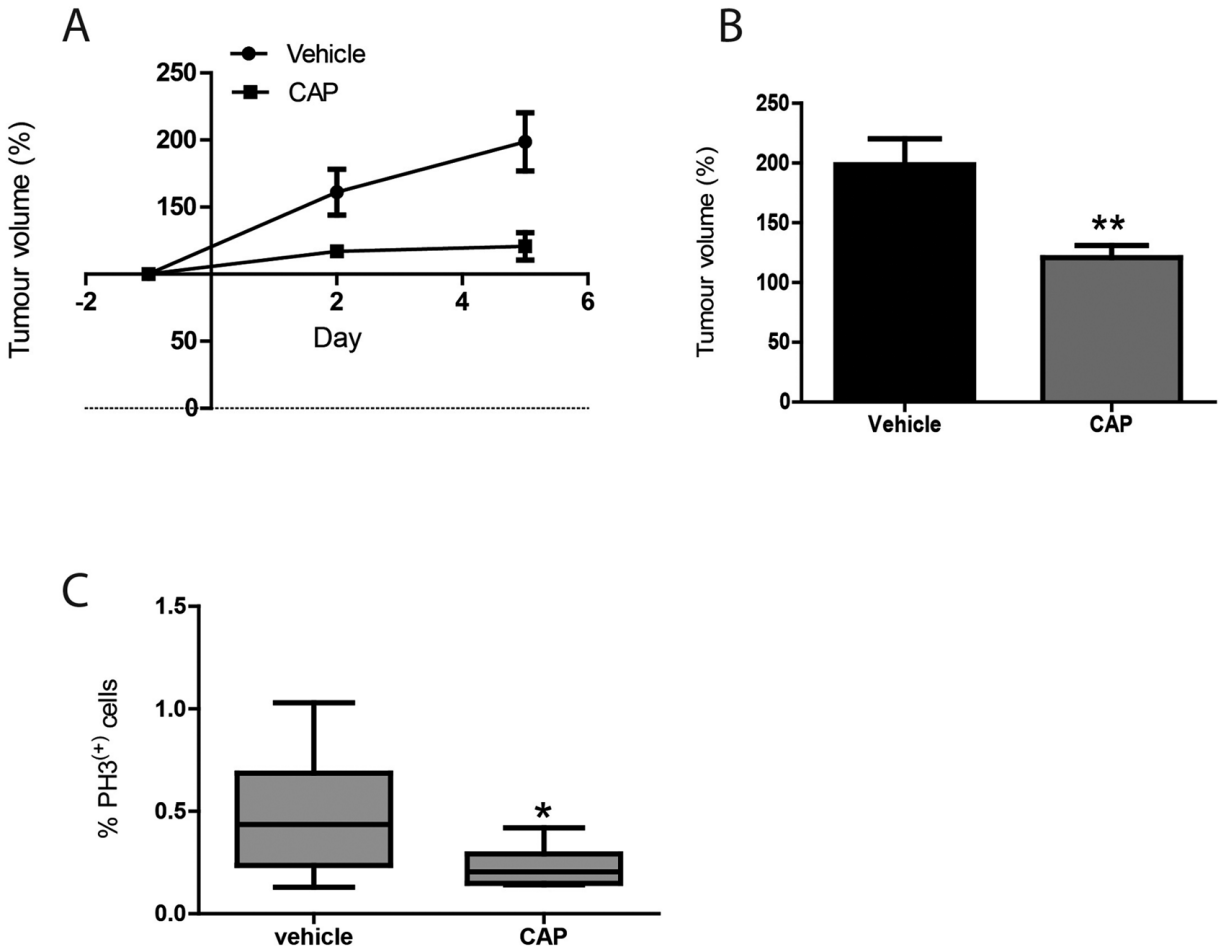
Anti-tumour effect of capecitabine in *in situ* PDAC tumours. (A) Short term capecitabine efficacy study in KPC mice with *in situ* PDAC, dosed with 755 mg/kg for 7 consecutive days. Tumour volumes for vehicle and CAP were measured by ultrasound and normalized to the tumour volume at the day of the enrolment in study (mean ± SEM, n = 6 per group). (B) Tumour volume at the endpoint as a percentage of the volume at the start, in KPC mice treated with vehicle or CAP (mean ± SEM, n = 6 in each group; **p<0.01). (C) Immunohistochemistry for phospho-histone H3 was quantified in PDAC tumours 2 h after the 7^th^ and last dose of CAP 755 mg/kg. Results are expressed as the mean ± SEM (n = 6 per group; *p = 0.027).

We then proceeded to a short term efficacy study in the *in situ* KPC PDAC model. KPC mice were treated with CAP at 755 mg/kg for 7 consecutive days and tumour volumes were monitored twice a week by 3D ultrasonography. Tumour growth was reduced in CAP-treated KPC mice compared to the vehicle-treated mice (Figure 3A). The volume of CAP- treated tumours was 121±10.2% compared to 199±21.8% in control (p<0.01; Figure 3B). Tumour tissue was collected 2 hours after the last dose of CAP. PH3 staining revealed less cells in mitosis in CAP-treated tumours compared to control (p<0.05; Figure 3C), suggesting growth arrest.

### The Effect of CAP Administration on KPC Mice Survival Compared to GEM

**Figure 4:**
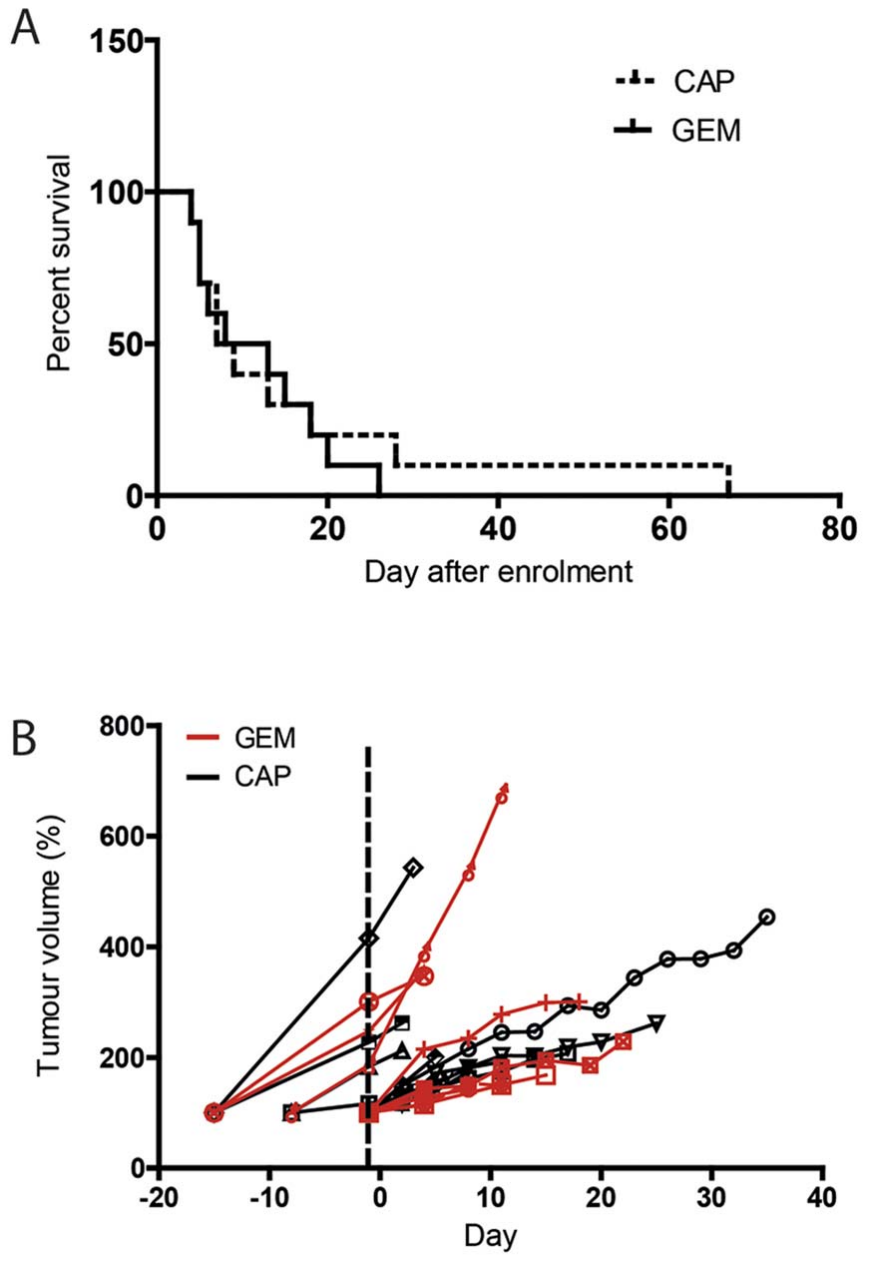
Comparison of CAP and GEM on KPC mice survival. (A): Kaplan-Meier curves for capecitabine (hatched) and gemcitabine (solid) survival. P = 0.61 Log-Rank test, Hazard Ratio = 0.78, 95%CI 0.29 to 2.06 (n = 10 per group). (B): Individual growth curves for CAP- (black) and GEM-treated tumours (red). Volumes were normalized to the volume at the day of the enrolment in the survival study.

Our pharmacokinetic data showed efficient 5-FU delivery and the short term CAP efficacy study data led us to compare CAP to GEM in a longer term study, using survival as the end-point. KPC mice were treated either with CAP at 755 mg/kg for 5 days per week or with 100 mg/kg GEM every 3 days. Results identified that survival was similar in the 2 groups; median survival 8 and 10.5 days for CAP and GEM respectively, P = 0.61 (Figure 4A). The longest survival was 67 days in the CAP group compared to 26 days in the GEM group. Furthermore, there was no difference in tumour growth between CAP- and GEM- treated groups (Figure 4B), suggesting similar efficacy of GEM and CAP in PDA.

### Effect of the GEMCAP Combination

**Figure 5:**
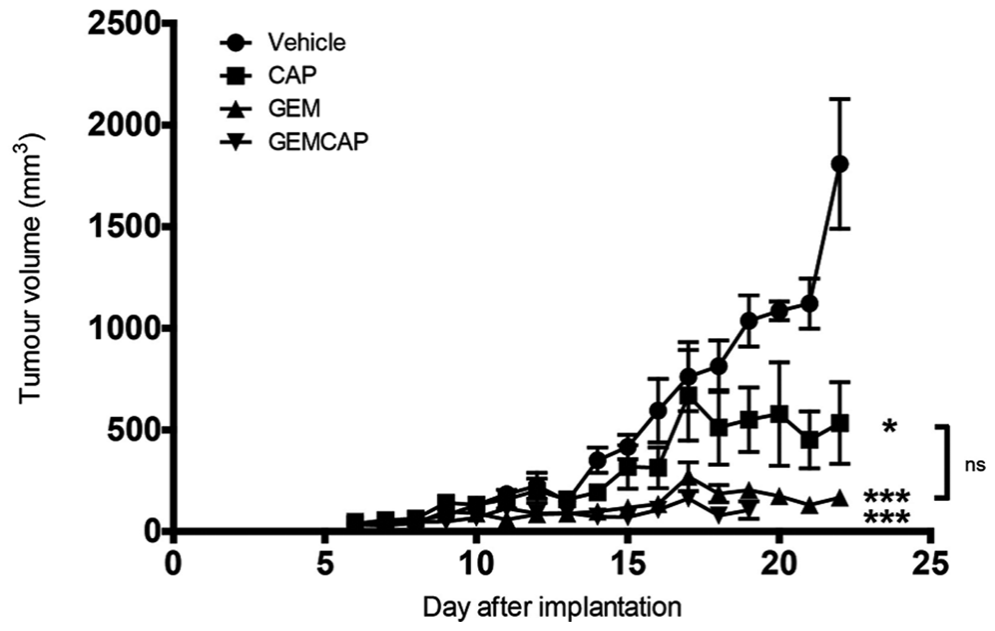
Anti-tumour effect of the GEMCAP combination in KPC allograft. GEMCAP combination efficacy in mice bearing KPC allograft tumours. Animals received vehicle or GEM every 3 days at 75 mg/kg or CAP at 539 mg/kg, 5 days per week or a combination of these two doses. Tumour volumes are represented as the mean ± SEM (n = 5 per group; *p<0.05, ***p<0.001 when compared to vehicle; ns: not significant).

Next, the combination of GEMCAP was investigated. In order to choose the appropriate doses, we first investigated the tolerability of the combination in mice bearing K8484 allografts. Mice were treated with GEM at 100 mg/kg every 3 days alone or in combination with CAP given 5 days per week at 755 mg/kg, 539 mg/kg or 378 mg/kg. All three combinations using full dose GEM induced marked gastrointestinal toxicity indicated by mouse body weight loss and the histology of the mouse small intestine: shortened villi with disrupted architecture and crypt loss (Supplemental Figure S1 D, F). Neither CAP 755 mg/kg or GEM 100 mg/kg alone showed any gastrointestinal toxicity (Figure S1, A – C) Therefore, we repeated the experiment with the same CAP doses combined with reduced GEM dose (75 mg/kg). The combination group treated with 755 mg/kg CAP showed early toxicity, confirmed by the small intestine histology (Figure S1, G). The combination with 539 mg/kg CAP (71% of full dose) exhibited no signs of toxicity over two cycles of treatment (11 days). Based on these results, we therefore used the safer doses of 539 mg/kg CAP and 75 mg/kg GEM to study the efficacy of the GEMCAP combination in mice with K8484 allografts. After 3 weeks, GEM significantly reduced tumour growth (p<0.001) compared to vehicle, as did CAP alone (p<0.05) (Figure 5). The difference observed between GEM and CAP alone was not significant (P>0.05). The GEMCAP combination significantly inhibited the tumour growth with tumour doubling time of 8.6±10.8 days (compared to 2.7±0.9 days in control) but this was not superior to GEM alone (tumour doubling time of 7.2±2.8 days) and not significantly different from CAP alone. During the 3^rd^ week of treatment, mice treated with the GEMCAP combination started to show toxicity (weight loss), compared to single agent treated mice but intestinal histology at the endpoint appeared normal (Figure S1, H and I).

These data, showing a lack of synergy of the combination, are consistent with those from *in vitro* cytotoxicity assays we performed on K8484 cells and human pancreatic cell lines (MiaPaCa-2 and Panc-1) dosed with a range of 5-FU and GEM combinations, where additive but not synergistic effects were observed (Figure S2). Taken together, our results showed additive cytotoxic effect *in vitro* but also additive toxicity *in vivo*. Therefore, GEMCAP does not lead to improved therapeutic index in mice when compared to GEM alone.

**Figure 6:**
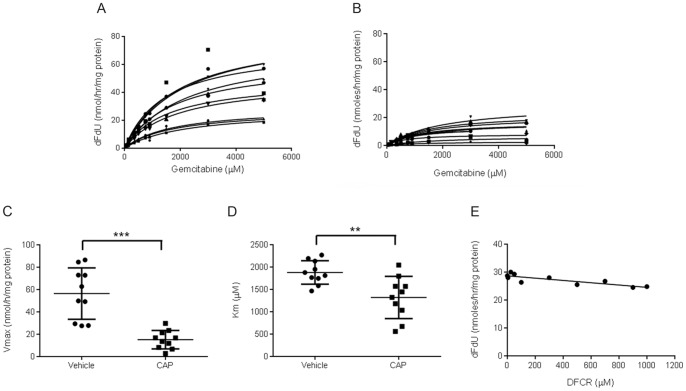
Cytidine deaminase enzyme activity in KPC allograft. CDA enzyme activity was determined in untreated (A) and CAP-treated (B) KPC allograft tumours using GEM as a substrate. The conversion rate of GEM into dFdU was measured in homogenates from individual tumours at the end of the CAP efficacy study presented in Figure 2, and the kinetic constants Vmax (C) and Km (D) were determined (n = 10 per group; **p<0.01, ***p<0.001). (E) *In vitro* competition assay performed on the enzyme extract from an untreated allograft tumour. A fixed GEM concentration of 1800 µM corresponding to the Km of CDA in the enzyme extract and DFCR concentrations ranging from 0 to 1200 µM were used.

We then investigated tumour CDA activity. Using GEM as a substrate, we determined the conversion rate of GEM into dFdU and the kinetic constants of CDA, Vmax and Km, in vehicle- and CAP-treated allograft tumours (Figure 6). Results showed reduced Vmax and Km in CAP-treated tumours (15.1±2.6 nmol/h/mg protein and 1320±147 µM respectively) compared to untreated tumours (56.3±7.2 nmol/h/mg protein and 1880±82 µM respectively; Figure 6C and 6D) suggesting that the gemcitabine metabolising capacity of CDA was decreased in transplanted PDAC tumours during CAP treatment. As the tumours were collected between 2 h and 4 h after the last dose of CAP, we wondered if the reduced activity was due to competition between the added GEM substrate and the DFCR already in the tumours. We therefore performed an *in vitro* competition assay using the enzyme extract from an untreated allograft tumour, a fixed GEM concentration of 1800 µM corresponding to the Km of CDA in the enzyme extract and DFCR concentrations ranging from 0 to 1200 µM. Results, presented in Figure 6E, showed minimal changes in the conversion rate of GEM into dFdU even in the presence of the highest concentration of DFCR. The LC-MS/MS analyses of CAP-treated tumours revealed that the highest DFCR concentrations was 511 µM, within the range tested in this competition assay, supporting that the observed decrease in CDA activity in CAP-treated tumours was not related to a substrate competition. This suggests that CAP treatment may downregulate CDA activity.

Because of the *in vivo* toxicity and the lack of enhanced effect of GEMCAP compared to GEM alone in the allografts, we did not test GEMCAP in KPC PDAC mice, whose health is less robust.

## Discussion

The studies presented here provide, for the first time, pre-clinical data on the pharmacokinetics and the efficacy of capecitabine in a genetically engineered mouse model of PDAC. We used the Kras^G12D^; Trp53^R172H^; Pdx1-Cre mouse model [12], because it recapitulates the clinical syndrome and histopathology of the human disease. Our pharmacokinetic data showed that, after oral administration, CAP undergoes extensive metabolism in plasma, liver and tumour. Concentrations of CAP metabolites in each of these compartments are consistent with those found in a human colon cancer xenograft [27]. They are also consistent with the previously reported distribution of the enzymes involved in the CAP metabolism in human and mouse [5], [27]. 5-FU was found at higher levels in tumour than in plasma from 30 min post administration and was undetectable in the liver confirming the tumour selective delivery of 5-FU after CAP administration also described by Ishikawa et al. [6]. The low 5-FU in plasma and liver may account for the low toxicity of CAP at the dose of 755 mg/kg: none of the mice showed signs of toxicity due to CAP treatment alone. We did not test higher doses because 755 mg/kg (2.1 mmol/kg) has been reported as the MTD [28].

We found equivalent concentrations of 5-FU in both KPC allograft and *in situ* PDAC tumours after CAP dosing. This finding was unexpected as impaired drug delivery has been reported in KPC tumours compared to allograft tumours after GEM dosing [18]. However, oral dosing of CAP extends systemic exposure time and may result in more sustained delivery to the tissues, leading to saturation in tissue compartments, than the bolus I.P. injection of GEM, which is cleared quickly. Impaired GEM delivery was attributed to poor vascularisation and the thickness of the stroma in PDAC tumours [18]. These factors should also affect 5-FU delivery. We also analysed the 5-FU content in different parts of the same *in situ* PDA tumour by mass spectroscopy and found homogeneous 5-FU concentrations, further supporting the hypothesis that prolonged administration leads to tissue saturation.

According to our experiments on GEM pharmacokinetics (not shown), the concentration of the active GEM metabolite, dFdCTP, was 2.35±1.21 µM in KPC allografts, 2 h after GEM dosing which is about 10 times less than the 5-FU concentration we found in these tumours at the same time after CAP dosing. Despite this difference, CAP induced a similar anti-tumour efficacy to GEM. This observation can be correlated to our *in vitro* data showing that in K8484 cells, the GEM IC_50_ is lower (1.4±1.8 nM) than the 5-FU IC_50_ (2.56±0.55 µM). It suggests that, although CAP and GEM were delivered to the tumour at therapeutically effective doses, higher doses of CAP are necessary to achieve the same anti-tumour effect than GEM.

CAP as a single agent was well tolerated in mice and induced similar effect to GEM on pancreatic tumour growth. This latter observation is supported by the results of a phase II clinical study in which 24% of patients with advanced PDAC and treated with CAP monotherapy had a clinical benefit response [29]. This clinical response rate was similar to the 23.8% reported for single agent GEM [30].

In view of clinical practice in the UK, we were keen to study the effect of the GEMCAP combination in our preclinical models. The combination of a nucleoside analogue with agents increasing nucleoside transporter (NT) expression at the cell surface has the potential for increased cytotoxicity. It has been shown that 5-FU depletes the endogenous intracellular nucleotide pools leading to increased NT abundance at the cell surface [31]–[33], and NT activity is a prerequisite for growth inhibition by GEM *in vitro* [34]. However, the *in vitro* data we have presented did not show any synergy between 5-FU and GEM. Rauchwerger et al. showed that 5-FU added prior to GEM, in Panc-1 cells, increased cell surface NT content and cytotoxicity to GEM but 5-FU doses used in that study (30 µM and 100 µM) were more than 100 fold higher than the 5-FU IC_50_ [33].

*In vivo,* significant toxicity was seen when GEM was combined with CAP and, as a result, reduced doses were required in combination, which showed no benefit in anti-tumour activity when compared to GEM alone. The meta-analysis performed by Heinemann et al. showed a significant survival benefit from the GEMCAP combination [2], based on phase III studies which individually showed a modest benefit [35] or only a trend in improving the overall survival and with an increase in toxicity for patients under GEMCAP treatment [36].

When GEM and CAP are combined, it is relevant to consider the role of cytidine deaminase (CDA), which is involved in the metabolism of both drugs. CDA plays a key role in the activation of CAP, through the deamination of DFCR to DFUR, and inactivates GEM through the deamination to dFdU [4], [5], [16], [17]. Therefore GEM and CAP metabolism may vary, according to the level of CDA activity in the relevant tissue. Transfection of human CDA into gastric cancer cell lines reduced their sensitivity to GEM both *in vitro* and *in vivo* [37] and forced expression of CDA increased the sensitivity of bladder cancer cells to DFCR but made them resistant to GEM *in vitro* and *in vivo* [38]. Our data suggests that the gemcitabine metabolising capacity of CDA was decreased in transplanted PDAC tumours during CAP treatment. Regulation of CDA activity by drug treatment has been described previously. Frese *et* *al*. demonstrated that nab-paclitaxel reduced the level of CDA expression in PDA tumours resulting in an increase of intra-tumoural GEM metabolites. In vitro experiments showed that CDA was inactivated through an induction of reactive oxygen species (ROS) [39]. 5-FU was shown to induce apoptosis through increase of intracellular ROS production in Jurkat cells, lung and colorectal cancer cells [40]–[42]. These data suggest that after treatment by CAP, 5-FU may induce ROS in KPC allografts, resulting in decreased activity of CDA.

A CAP-induced decrease in CDA activity could lead to improved anti-tumour efficacy with the GEMCAP combination, but also may explain the increased toxicity associated with the GEMCAP combination in our studies as, in mice, high CDA activity is seen in the intestine [43]. Supporting this hypothesis, patients with CDA deficiency experience severe toxicities after GEM-based chemotherapies [14].

In summary, our pre-clinical data support the use of CAP in the treatment of PDAC, although we were unable to demonstrate, in these models, a benefit for the combination with GEM, because increased toxicity forced dose reduction of both agents. In view of the activity seen as a single agent, CAP could be considered as an alternative to GEM in future combination treatment strategies, where a fluoropyrimidine may be a more rational partner for novel agents than GEM.

## Supporting Information

Figure S1 **Histology of small intestine from GEMCAP- treated mice.** H&E stained sections showing the small intestine architecture after administration of vehicle (A), CAP 755 mg/kg (B), GEM 100 mg/kg (C), GEM 100 mg/kg and CAP 755 mg/kg (D), GEM 100 mg/kg and CAP 539 mg/kg (E), GEM 100 mg/kg and CAP 378 mg/kg (F), GEM 75 mg/kg and CAP 755 mg/kg (G), GEM 75 mg/kg and CAP 539 mg/kg (H) and GEM 75 mg/kg and CAP 378 mg/kg (I).
(TIF)

Figure S2 **Evaluation of GEMCAP combination in mouse and human pancreatic cancer cell lines** ***in vitro.*** K8484 (A), Panc-1 (B) and MIAPaCa-2 (C) cells were exposed to combinations of concentrations of GEM (0–300 nM) and 5-FU (0–30,000 nM) for 72 h then SRB staining was used to determine the % of growth compared to solvent control (1). Predicted growth inhibitions were calculated using the Bliss Additivity model with the single agent data (2) and then subtracted from the experimental data to give a difference value for each combination (3). The numbers in each square are the mean and standard deviation of 3 replicates and each square is colour-coded according to the heatmap of the difference values (scale shown on the right). Negative difference values, shown in blue would denote synergy and positive difference values, shown in red, would denote antagonism.
(TIF)
