# Supplementary figures and images for: Anti-Tumour Efficacy of Capecitabine in a Genetically Engineered Mouse Model of Pancreatic Cancer
Source: PLoS One. 2013 Jun 28;8(6):e67330. doi: 10.1371/journal.pone.0067330 (PMC3696095; doi:10.1371/journal.pone.0067330)

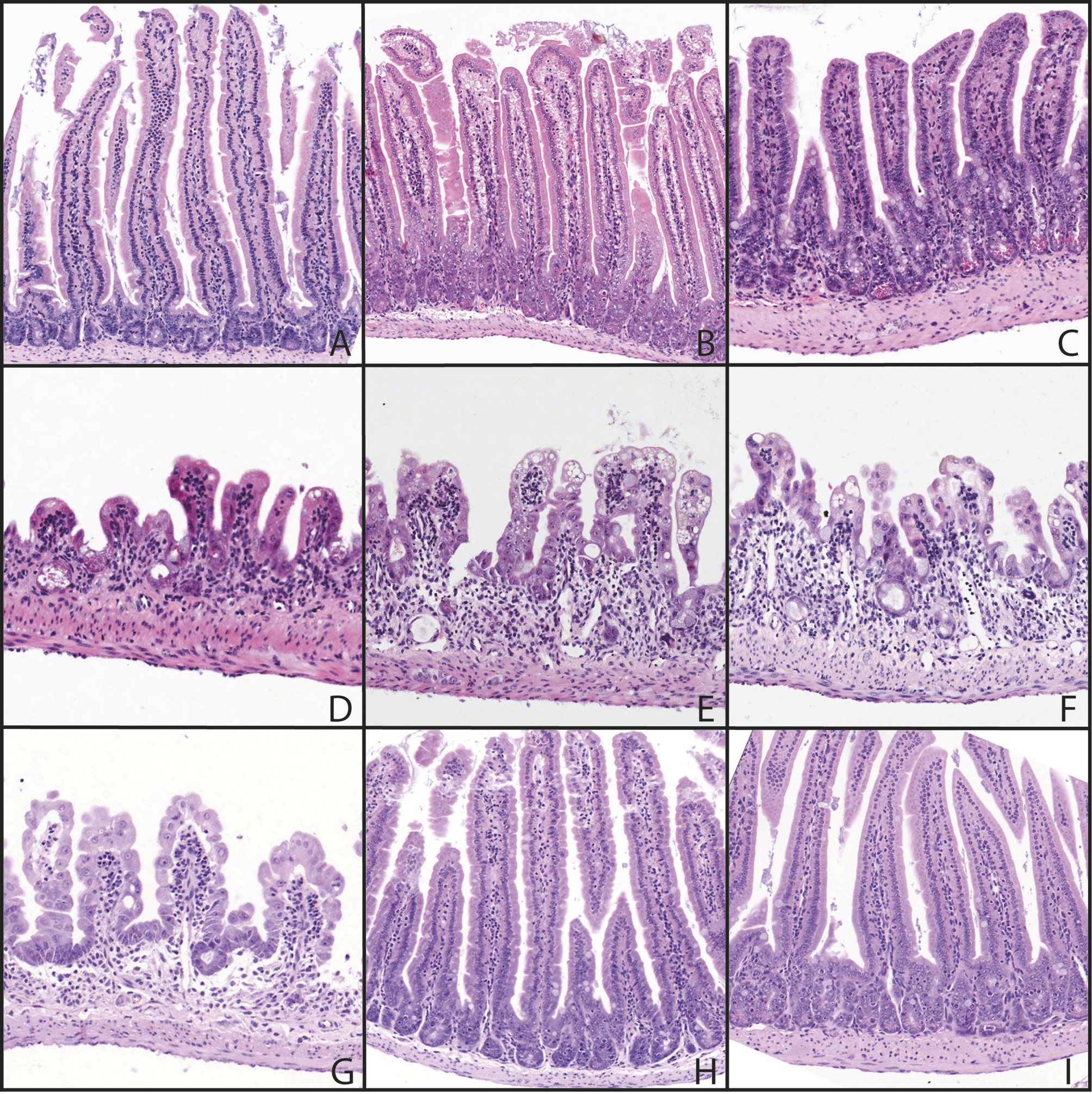

Supplement: Figure S1 — Histology of small intestine from GEMCAP- treated mice. H&E stained sections showing the small intestine architecture after administration of vehicle (A), CAP 755 mg/kg (B), GEM 100 mg/kg (C), GEM 100 mg/kg and CAP 755 mg/kg (D), GEM 100 mg/kg and CAP 539 mg/kg (E), GEM 100 mg/kg and CAP 378 mg/kg (F), GEM 75 mg/kg and CAP 755 mg/kg (G), GEM 75 mg/kg and CAP 539 mg/kg (H) and GEM 75 mg/kg and CAP 378 mg/kg (I). (TIF) [file pone.0067330.s001.tif]

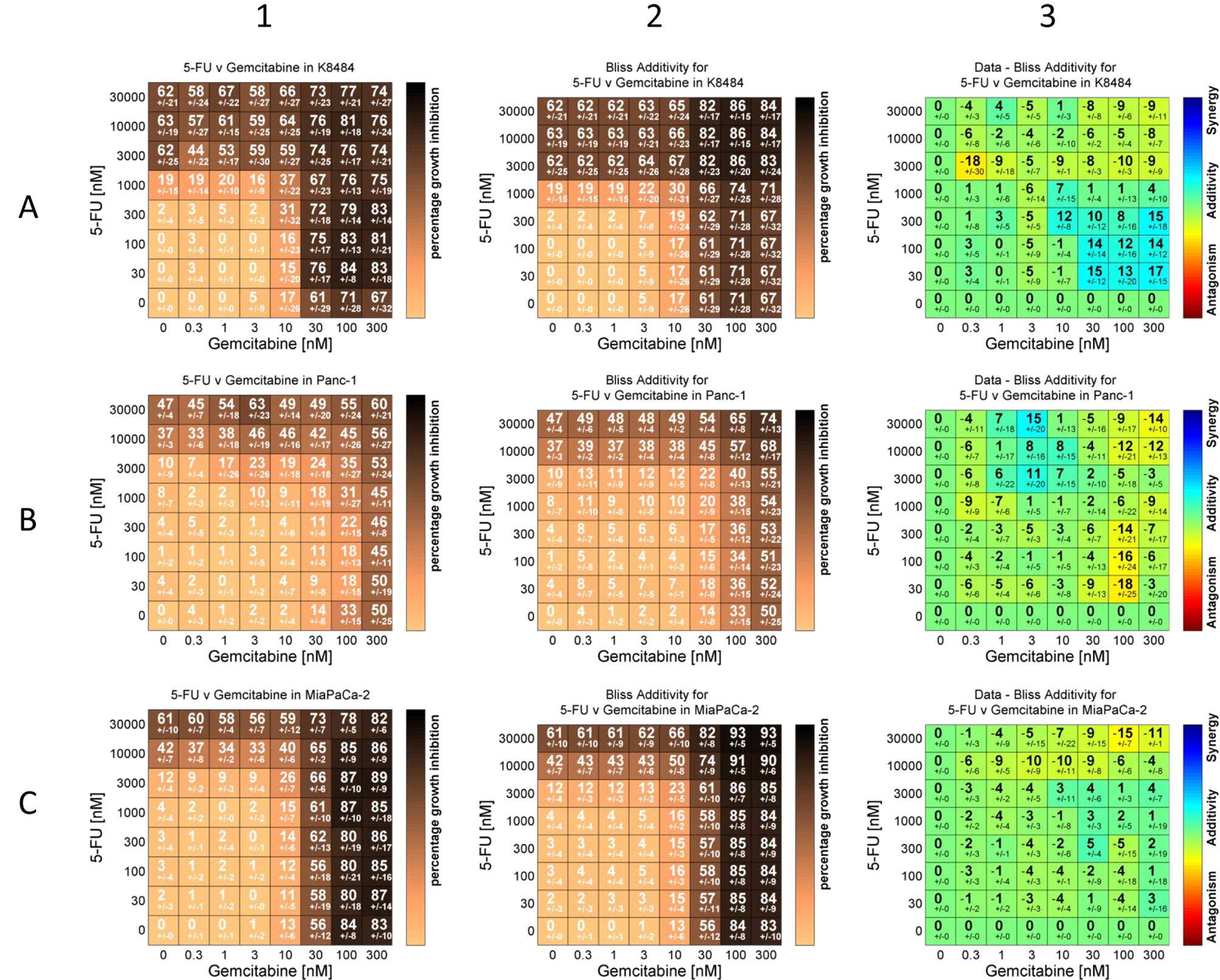

Supplement: Figure S2 — Evaluation of GEMCAP combination in mouse and human pancreatic cancer cell lines in vitro. K8484 (A), Panc-1 (B) and MIAPaCa-2 (C) cells were exposed to combinations of concentrations of GEM (0–300 nM) and 5-FU (0–30,000 nM) for 72 h then SRB staining was used to determine the % of growth compared to solvent control (1). Predicted growth inhibitions were calculated using the Bliss Additivity model with the single agent data (2) and then subtracted from the experimental data to give a difference value for each combination (3). The numbers in each square are the mean and standard deviation of 3 replicates and each square is colour-coded according to the heatmap of the difference values (scale shown on the right). Negative difference values, shown in blue would denote synergy and positive difference values, shown in red, would denote antagonism. (TIF) [file pone.0067330.s002.tif]
